# Distinct Responses of *Mycobacterium smegmatis* to Exposure to Low and High Levels of Hydrogen Peroxide

**DOI:** 10.1371/journal.pone.0134595

**Published:** 2015-07-30

**Authors:** Xiaojing Li, Jun Wu, Jiao Han, Yongfei Hu, Kaixia Mi

**Affiliations:** 1 CAS Key Laboratory of Pathogenic Microbiology and Immunology, Institute of Microbiology, CAS, Beijing, 100101, China; 2 Beijing Key Laboratory of Microbial Drug Resistance and Resistome, Beijing 100101, Beijing, China; Indian Institute of Science, INDIA

## Abstract

Hydrogen peroxide (H_2_O_2_) is a natural oxidant produced by aerobic organisms and gives rise to oxidative damage, including DNA mutations, protein inactivation and lipid damage. The genus *Mycobacterium* utilizes redox sensors and H_2_O_2_ scavenging enzymes for the detoxification of H_2_O_2_. To date, the precise response to oxidative stress has not been fully elucidated. Here, we compared the effects of different levels of H_2_O_2_ on transcription in *M*. *smegmatis* using RNA-sequencing. A 0.2 mM H_2_O_2_ treatment had little effect on the growth and viability of *M*. *smegmatis* whereas 7 mM H_2_O_2_ was lethal. Analysis of global transcription showed that 0.2 mM H_2_O_2_ induced relatively few changes in gene expression, whereas a large proportion of the mycobacterial genome was found to be differentially expressed after treatment with 7 mM H_2_O_2_. Genes differentially expressed following treatment with 0.2 mM H_2_O_2_ included those coding for proteins involved in glycolysis-gluconeogenesis and fatty acid metabolism pathways, and expression of most genes encoding ribosomal proteins was lower following treatment with 7 mM H_2_O_2_. Our analysis shows that *M*. *smegmatis* utilizes the sigma factor MSMEG_5214 in response to 0.2 mM H_2_O_2_, and the RpoE1 sigma factors MSMEG_0573 and MSMEG_0574 in response to 7 mM H_2_O_2_. In addition, different transcriptional regulators responded to different levels of H_2_O_2_: MSMEG_1919 was induced by 0.2 mM H_2_O_2_, while high-level induction of DevR occurred in response to 7 mM H_2_O_2_. We detected the induction of different detoxifying enzymes, including genes encoding KatG, AhpD, TrxB and Trx, at different levels of H_2_O_2_ and the detoxifying enzymes were expressed at different levels of H_2_O_2_. In conclusion, our study reveals the changes in transcription that are induced in response to different levels of H_2_O_2_ in *M*. *smegmatis*.

## Introduction

The genus *Mycobacterium* includes pathogens, such as *Mycobacterium tuberculosis* and non-pathogenic microorganisms, such as *Mycobacterium smegmatis*. Mycobacteria are able to respond to and survive under different stresses [1]. Hydrogen peroxide (H_2_O_2_) is a natural stressor that is produced by aerobic organisms and leads to oxidative damage, such as DNA mutations, protein inactivation and lipid damage [2]. In addition, when *M*. *tuberculosis*, the pathogen which causes human tuberculosis (TB), infects a host, the production of H_2_O_2_ is an important innate defense mechanism against infection. As a successful pathogen, *M*. *tuberculosis* has evolved redox sensors and H_2_O_2_ scavenging enzymes for the detoxification of H_2_O_2_ damage [3,4], but the precise response to H_2_O_2_ has not been fully elucidated. A number of studies have shown that *M*. *tuberculosis* contains several regulators that respond to H_2_O_2_ and several enzymes that detoxify H_2_O_2_ damage [5–7]. A recent study has reported different transcriptional profiles in *M*. *tuberculosis* in response to different H_2_O_2_ concentrations [7]. However, the transcriptional response of *M*. *smegmatis* to different concentrations of H_2_O_2_ has yet to be explored. A greater understanding of the differences between pathogenic *M*. *tuberculosis* and nonpathogenic *M*. *smegmatis* in their response to H_2_O_2_ will help us to understand the pathogenesis of *M*. *tuberculosis*.

Transcriptional regulation in response to H_2_O_2_ in the *Mycobacteria* is complex compared to that in *Bacillus* or *Escherichia coli*. *M*. *tuberculosis* has 13 sigma factors, and *M*. *smegmatis* has 28 sigma factors [8,9], of which SigE, SigH, SigL and SigF play important roles in oxidative stress [3]. As classical transcriptional regulators such as OxyR, FNR and FixL are absent in *M*. *tuberculosis*, alternative transcriptional regulators have been suggested to be involved in oxidative stress, including FurA [10], IdeR [11], CarD [12], and the WhiB proteins [3]. In addition to transcriptional regulators involved in the response to H_2_O_2_, the signal transduction network including two-component systems, one-component systems, and serine/threonine kinases, is also involved in relaying and orchestrating the response to H_2_O_2._ *M*. *tuberculosis* encodes 11 serine/threonine kinases (STKs), of which PknB, PknF, and PknG have been shown to be involved in the oxidative stress response [13–15]. Park et al. showed that PknB phosphorylates both SigH and its anti-sigma factor RshA and causes its release from the complex of SigH and RshA. The phosphorylated SigH then regulates the response to oxidative stress [14]. Similar to PknB, PknD was shown to phosphorylate anti-anti-sigma factor Rv0516c and then to activate Rv0516c, which changes the expression of the SigF regulon [13]. Moreover, *M*. *tuberculosis* produces many enzymes that scavenge H_2_O_2_. Mycobacterial KatG is a multifunctional heme-dependent catalase-peroxidase-peroxynitritase [16] and efficiently protects *Mycobacterium* from reactive oxygen species damage [17]. KatG is the target of the first-line drug isoniazid (INH) and is responsible for the conversion of the prodrug INH into active INH [18]. Clinical strains with decreasing KatG activity showed higher levels of AhpC [19], suggesting that AhpC contributes to defense against oxidative stress. The metabolic enzyme complex with Lpd, SucB, AhpC, and AhpD, is also involved in antioxidant defense [20]. Thiol-dependent peroxidase Tpx is an antioxidant protein against oxidative stress [21]. Voskuil et al. recently investigated whole genome expression in response to different levels of oxidative stress and showed that many genes related to oxidative stress are induced concurrently with the dormancy regulon at high concentrations of H_2_O_2_ [7].

In this study, we compared the effects of different H_2_O_2_ levels on transcription in *M*. *smegmatis* using RNA-sequencing. We show that low levels (0.2 mM) of H_2_O_2_ have little effect on the growth and viability of *M*. *smegmatis* whereas high levels (7 mM) of H_2_O_2_ are bactericidal. Relatively few changes in gene expression were observed on exposure to 0.2 mM H_2_O_2_ while a large munber of differentially expressed genes were induced after treatment with 7 mM H_2_O_2_. Some differentially expressed genes involved in the glycolysis-gluconeogenesis and fatty acid metabolic pathways were induced by 0.2 mM H_2_O_2_, and the expression of genes encoding ribosomal proteins was lower after treatment with 7 mM H_2_O_2._ Our analysis also identified differences in the sigma factors, transcriptional regulators, and detoxifying enzymes that are expressed in response to treatment with 0.2 mM and 7 mM H_2_O_2_.

## Materials and Methods

### Bacterial strains and culture conditions

Liquid cultures of the *M*. *smegmatis* mc^2^155 strain were grown in Middlebrook 7H9 medium (Becton Dickinson) supplemented with 0.2% (v/v) glycerol (Beijing Modern Eastern Finechemical), 0.05% Tween 80 (v/v) (Sigma) and 10% ADS (albumin, dextrose, and saline). Middlebrook 7H10 medium (Becton Dickinson) supplemented with 10% ADS and 0.2% (v/v) glycerol was used as the solid medium for *M*. *smegmatis* growth.

### Response of the *M*. *smegmatis* mc^2^155 strain to H_2_O_2_ stress

Log phase cultures (OD_600_ of 0.8–1.0) of *M*. *smegmatis* mc^2^155 were diluted 1:100 into 7H9 media and cultured for approximately 12 hours until the OD_600_ reached 0.3. Re-inoculated cells were then treated with the indicated concentrations of H_2_O_2_ (0, 0.2 and 7 mM) for periods of 30 min or 3 h, and surviving cells were grown on 7H10 media. Cells were collected after 30 min of 0.2 mM or 7 mM H_2_O_2_ treatment, and total RNA was isolated from each sample and compared by RNA-sequencing to RNA from untreated cells that were prepared simultaneously.

### RNA isolation for RNA-sequencing

Fifty milliliters of bacterial culture (OD_600_ of ~ 0.3) was collected and total RNA was isolated using FastPrep Purification kits (MP Bio) according to the manufacturer’s instructions. Construction and sequencing of the cDNA libraries of the various mycobacterial strains was performed by BGI-Shenzhen (China). Briefly, total RNA from treated mc^2^155 strains was treated using a Ribominus Transcriptome Isolation Kit (Thermo Fisher Scientific) to remove rRNA contaminations. NEXTflex RNA Fragmentation Buffer (Bioo Scientific) was added to separate the mRNA into short fragments. Using these short fragments as templates, random hexamer-primers were used to synthesize the first strand of cDNA. The second strand of cDNA was synthesized using buffer, dNTPs, RNase H and DNA polymerase I. The short fragments were purified with a QIAQuick PCR extraction kit (QIAGEN) and resolved with EB buffer for end reparation and addition of poly(A). The short fragments were subsequently connected with sequencing adaptors. For amplification by PCR, we selected suitable fragments as templates, based on results of agarose gel electrophoresis. The library was then sequenced using Illumina HiSeq 2000. Clean reads were mapped to the reference genome and the gene sequences using SOAP2 [22]. The RNA-sequencing dataset obtained has been submitted to ArrayExpress under the accession number E-MTAB-3594.

### RNA-sequencing data analysis

The raw data were filtered to 1) remove reads with adaptors, 2) remove reads with more than 10% of unknown nucleotides, 3) remove low quality reads (in which more than half of the base quality scores were less than 5). The resulting cleaned paired-end reads were mapped to the *M*. *smegmatis* mc^2^155 reference genome (NC 008596.1) using SOAP2. Mismatches of no more than 5 bases were allowed in the alignment. We performed statistical analysis in read alignments on the genome and genes for each sample. Randomness of the mRNA/cDNA fragmentation was evaluated using the reads distribution of reference genes.

Gene expression was calculated according to the RPKM method (reads per kilobase of exon model per million mapped reads) [23], and eliminated the biases influence of different gene length and sequencing difference using the TPM method [24]. We calculated the ratio of each gene between samples and identified genes differentially expressed between two samples using "the significance of digital gene expression profiles" [25] based on the criteria FDR ≤ 0.001 and a fold change larger than 4. STRING (9.1) [26] was used to analyze the interactions of differentially expressed genes and functional and pathway enrichment analysis. A p-value less than 0.05 was used as a threshold to indicate significant enrichment.

### Quantitative PCR of selected genes

Log phase cultures (OD_600_ = 0.8–1.0) of all the tested strains were diluted 1:100 in 7H9 media. The strains were cultured until the OD_600_ reached 0.3 and then divided into control and treatment groups. In the treatment group, the cells were treated with 0.2 or 7 mM H_2_O_2_ for 30 min and then collected by centrifugation at 12,000 x g. Bacterial pellets were resuspended in TRIzol (Invitrogen, USA), and RNA was purified according to the manufacturer’s instructions. cDNA was synthesized using the SuperScript III First-Strand Synthesis System (Invitrogen, USA). Quantitative real-time PCR (qRT-PCR) was performed in a Bio-Rad iCycler using a 2x SYBR real-time PCR pre-mix (Takara Biotechnology Inc., Japan). The following cycling program was used: 95°C for 1.5 min followed by 40 cycles of 95°C for 10 s, 60°C for 15 s, and 72°C for 15 s, followed by 72°C for 6 min. The *M*. *smegmatis rpoD* gene encoding the RNA polymerase sigma factor SigA was selected as a reference gene for normalizing gene expression. The 2^-ΔΔCT^ method was used [27] to evaluate relative gene expression in the different strains and/or different treatments. All primers used are listed in S1 Table.

### Statistical analysis

All statistical analyses were performed using GraphPad Prism 5.0c. Significant differences in the data were determined using t-tests.

## Results and Discussion

### Effects of H_2_O_2_ on growth and viability

Hydrogen peroxide (H_2_O_2_) is a natural oxidant produced by aerobic organisms and can lead to oxidative damage, such as DNA mutations, protein inactivation and drug resistance [2]. In addition, increasing levels of toxic H_2_O_2_ in the infected host is an important defensive mechanism against invading pathogens. Resistance to H_2_O_2_ might increase bacterial survival in mycobacterial-infected macrophages. A previous study from our lab showed that increased resistance to H_2_O_2_ in a mutant strain of *M*. *smegmatis* could lead to higher survival in infected macrophages [28]. *M*. *tuberculosis* can persist in macrophages for decades, partly because it possesses many regulators that respond to H_2_O_2_ and many enzymes that detoxify H_2_O_2_ [3,7]. A recent study analyzed genome-wide changes in gene expression in response to different levels of oxidative and nitrosative stresses in *M*. *tuberculosis* [7]. Responses to oxidative and nitrosative stresses were compared and their results revealed a common genetic response used by *M*. *tuberculosis* in response to these stresses. This study demonstrated that analyzing global transcription levels can help us understand the molecular mechanisms underlying the response of bacteria to H_2_O_2_. Here, we examined the global transcriptional response of *M*. *smegmatis* to different levels of H_2_O_2_ using RNA-sequencing.

**Fig 1:**
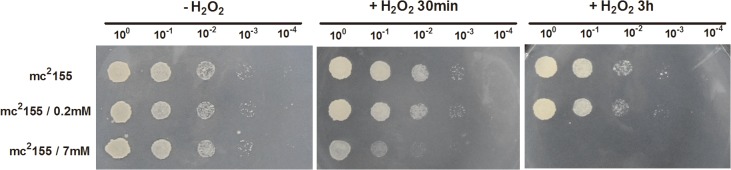
The effect of H_2_O_2_ stress on the survival of *M*. *smegmatis*. The panel represents serial dilutions (1:10) of mc^2^155 cultures treated with 0.2 mM or 7 mM H_2_O_2_ for either 30 min or 3 hour. Three microliters of diluted *M*. *smegmatis* cultures were spotted onto solid 7H10 medium. Images shown are representative of at least 3 experiments.

Bacteria are most sensitive to environmental stresses at the early logarithmic phase [29]. We therefore chose to treat the *M*. *smegmatis* strain mc^2^155 with H_2_O_2_ when bacteria reached the early logarithmic phase of growth (optical density, OD_600_ of ~ 0.3). We have previously reported that, under experimental conditions in our laboratory, the MIC to H_2_O_2_ in *M*. *smegmatis* is 0.039 mM [28] and that of *M*. *tuberculosis* is 1mM. The ratio of the MIC to H_2_O_2_ of *M*. *smegmatis* to *M*. *tuberculosis* is ~26. In order to compare the response of *M*. *smegmatis* and *M*. *tuberculosis* to H_2_O_2_, we used concentrations of H_2_O_2_ comparable to those used in Voskuil et al [7]. Here we used an H_2_O_2_ concentration of 7mM for *M*. *smegmatis* to correspond to the 200 mM H_2_O_2_ treatment used by Voskuil et al. in *M*. *tuberculosis*. Similarly, the 0.2 mM H_2_O_2_ treatment used here corresponded to the ~ 5 mM H_2_O_2_ treatment used by Voskuil et al in *M*. *tuberculosis*. When the *M*. *smegmatis* mc^2^155 strain reached the early logarithmic phase, bacterial cultures were exposed to two different H_2_O_2_ concentrations, namely 0.2 mM or 7 mM for either 30 min or 3 h, and cultures were then collected and spotted onto 7H10 media. As shown in Fig 1, no growth differences were detected between the bacteria treated with 0.2 mM for 30 min or 3 h and untreated control bacteria, indicating that exposure to 0.2 mM H_2_O_2_ had little effect on bacterial growth and viability. In contrast, when cultures were treated with 7 mM H_2_O_2_, we observed that exposure to 7 mM H_2_O_2_ resulted in a significant decrease in cell survival (Fig 1), indicating that 7 mM H_2_O_2_ has a bactericidal effect in *M*. *smegmatis*. Moreover, the genes induced in response to H_2_O_2_ (5–10 mM) in the Voskuil study were also found to be induced in *M*. *tuberculosis* during infection of activated macrophages[30], indicating that a level of 5–10 mM H_2_O_2_ is similar to that experienced by bacteria within infected macrophage. Here, the response of 7 mM of H_2_O_2_ used to examine the *M*. *smegmatis* response might suggest the response of bacteria within infected macrophage.

The following experiments were performed to compare the transcriptional response of *M*. *smegmatis* exposed to 0.2 mM H_2_O_2_ or 7 mM H_2_O_2_ for 30 min when bacterial growth had reached an OD_600_ of 0.3.

### Expression profiles of *M*. *smegmatis* in response to different levels of H_2_O_2_

Transcriptional reprogramming is a critical step in bacterial responses to various stress factors to ensure their survival. We therefore examined changes in mRNA expression following treatment with H_2_O_2_ using RNA-sequencing. mRNA samples from of *M*. *smegmatis* mc^2^155 with or without H_2_O_2_ treatment were prepared as described in the “Materials and Methods”.

RNA-sequencing mapping statistics showed that approximately 96% of the sequencing reads could be mapped to the *M*. *smegmatis* reference genome (NC_008596.1) (Table 1). The percentage of unique mapped reads for untreated mc^2^155, mc^2^155 treated with 0.2 mM H_2_O_2_ and mc^2^155 treated with 7 mM H_2_O_2_ were 92.34%, 98.28% and 98.17%, respectively and the number of reads mapped was 6089174, 6390446 and 6548357, respectively.

**Table 1:** RNA-sequencing mapping statistics.

Sample name	mc^2^155		mc^2^155 / 0.2 mM		mc^2^155/ 7 mM	
	Reads number	Percentage	Reads number	Percentage	Reads number	Percentage
Total reads	6594442	100.00%	6502552	100.00%	6670090	100.00%
Total base pairs	593499780	100.00%	585229680	100.00%	600308100	100.00%
Total Mapped Reads	6089174	92.34%	6390446	98.28%	6548357	98.17%
Perfect match	4382586	66.46%	5499445	84.57%	5525430	82.84%
≤ 5bp mismatch	1706588	25.88%	891001	13.70%	1022927	15.34%
Unique match	5818976	88.24%	6065452	93.28%	6171788	92.53%
Multi-position match	270198	4.10%	324994	5.00%	376569	5.65%
Total unmapped reads	505268	7.66%	112106	1.72%	121733	1.83%

**Fig 2:**
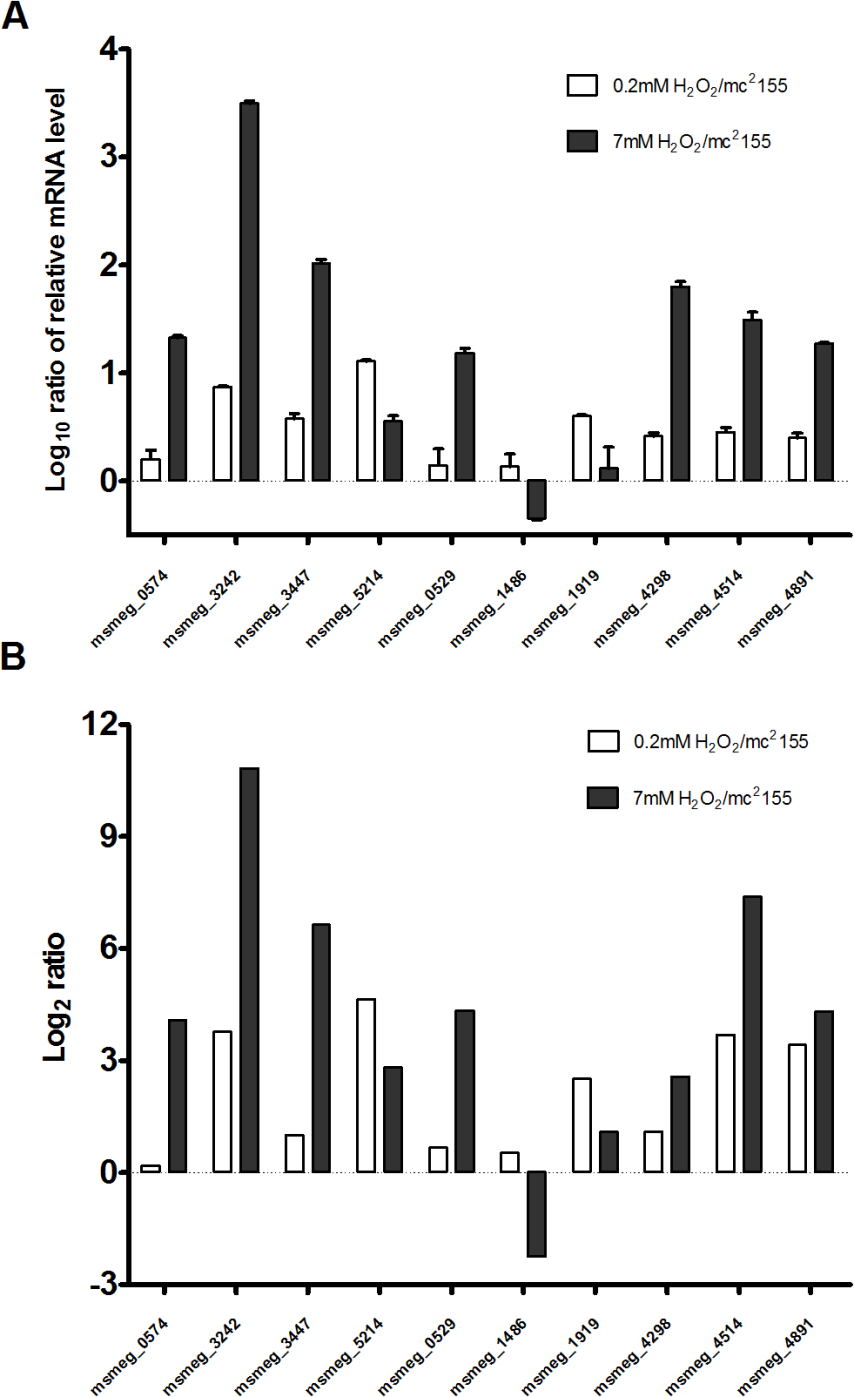
Quantitative RT-PCR validation of RNA-sequencing results. (A) Quantitative RT-PCR analysis of the mRNA expression of genes differentially expressed after treatment with different levels of H_2_O_2_. *M*. *smegmatis* cultures were treated with 2 mM or 7 mM H_2_O_2_ for 30 min before extraction of RNA for qRT-PCR. The data represent 3 independent experiments. (B) Fold changes of selected genes differentially expressed genes after treatment with 0.2 mM and 7 mM H_2_O_2_ obtained by the RNA-sequencing.

Fully annotated data are presented in S2–S4 Tables. Genes were considered to be significantly differentially expressed if their changes in expression were > 4-fold greater compared to the non-treated wild type mc^2^155 strain, with a false discovery rate (FDR) corrected P-value of < 0.01. To confirm the results obtained from the RNA-sequencing analysis (Fig 2B), several induced genes were examined by quantitative RT-PCR (qRT-PCR). In three independent experiments, total RNA was isolated from *M*. *smegmatis* exposed to 0.2 mM or 7 mM H_2_O_2_ for 30 min and relative levels of expression were analyzed by qRT-PCR. Results were consistent with those obtained from RNA-sequencing results, confirming the validity of our approach. For example, *msmeg_0574* exhibited a 21.17 ± 1.11-fold enhancement when induced by 7 mM H_2_O_2_, but little enhancement (1.61 ± 0.31-fold) when induced by 0.2 mM H_2_O_2_ (Fig 2A), consistent with RNA-sequencing results (Fig 2B). In addition, *msmeg_3242* exhibited a 3116.9 ± 182.8-fold enhancement when induced by 7 mM H_2_O_2_ and a 7.43 ± 0.11-fold enhancement when induced by 0.2 mM H_2_O_2_ (Fig 2A)_._ The relative expression levels of the genes we chose to test under 0.2 mM and 7 mM H_2_O_2_ were also consistent with RNA-sequencing results (Fig 2). In summary, these results support the fidelity of the RNA-sequencing results for the analysis below.

**Fig 3:**
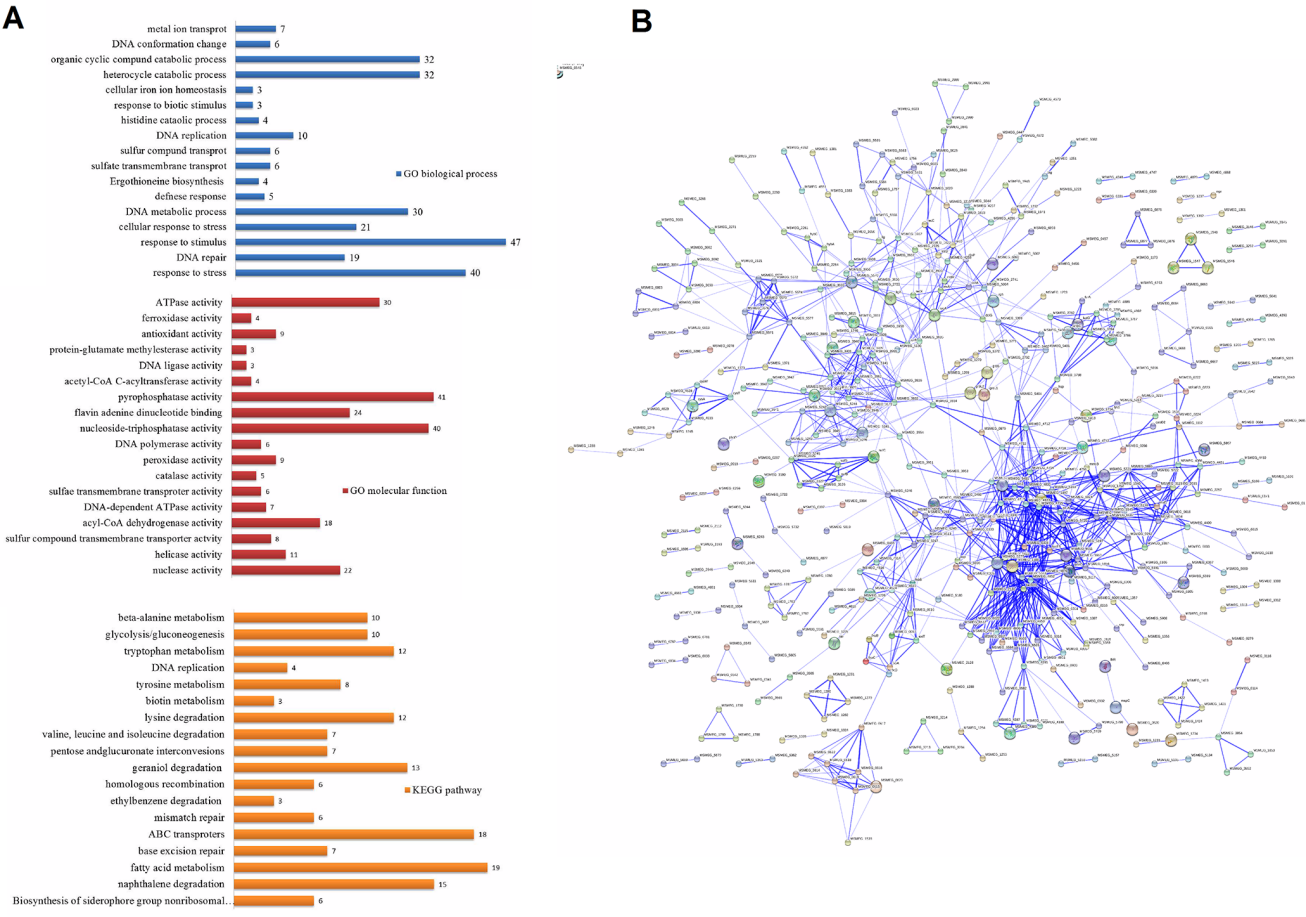
Overview of the differential expression profiles in response to 0.2 mM H_2_O_2_ in *M*. *smegmatis*. (A) Enrichment analysis. The differently colored bars indicate the gene number for the enrichment of the annotations. (B) Interaction network of the differentially expressed genes of *M*. *smegmatis* induced by 0.2 mM H_2_O_2_ using STRING (9.1) at confidence scores ≥ 0.4. The network is enriched among the 634 differentially expressed genes and 111 interactions were observed (p value = 0).

**Fig 4:**
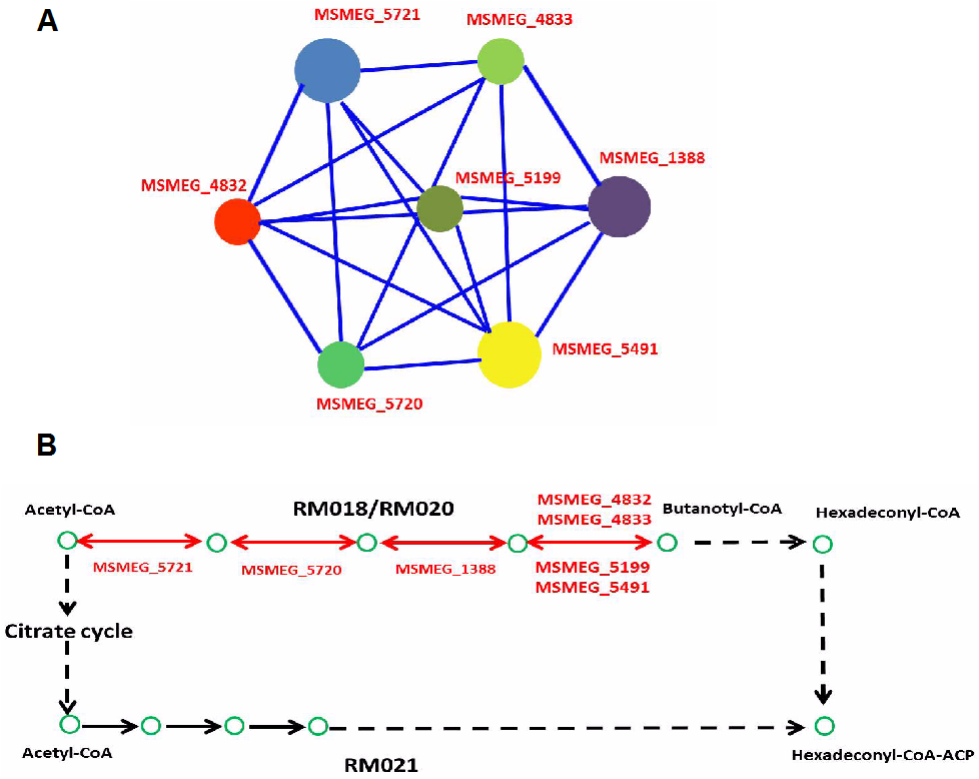
Connected network of the enriched differentially expressed genes following exposure to 0.2 mM H_2_O_2_. (A) Connected network of enriched differentially expressed genes involved in fatty acid metabolism (RM018 and RM020). (B) Partial fatty acid metabolism in *M*. *smegmatis*. Genes expressed differentially after 0.2 mM H_2_O_2_ treatment assigned to RM018 and RM020 are marked in red.

Upon exposure to 0.2 mM H_2_O_2,_ there were 303 up-regulated genes and 331 down-regulated genes. Genes differentially expressed in the mc^2^155 strain on treatment with 0.2 mM H_2_O_2_ were significantly enriched for several GO biological processes, including response to stress (p = 1.44 x 10^−10^), DNA repair (p = 2.87 x 10^−5^), and ergothioneine biosynthesis (p = 1.18 x 10^−2^) when compared to the untreated mc^2^155 strain (Fig 3A). In GO molecular function categories, we found that genes differentially expressed after treatment with 0.2 mM H_2_O_2_ were significantly enriched for nuclease activity (p = 2.49 x10^-4^), helicase activity (p = 4.55 x 10^−2^), and sulfur compound transmembrane transporter activity (p = 4.55 x 10^−2^) when compared to the untreated mc^2^155 strain (Fig 3A). As H_2_O_2_ causes DNA damage, genes involved in DNA repair (listed in Table 2) were induced upon exposure to 0.2 mM H_2_O_2_. Induction of RecA, AlkA, and DNA helicase by H_2_O_2_ was also found in the *M*. *tuberculosis* study [7]. *M*. *tuberculosis* RecA is involved in nucleotide excision, recombination and the SOS response [31]. In *M*. *smegmatis*, RecA is induced by DNA damage and is a key regulator element of the SOS response [32]. In *M*. *tuberculosis*, *dnaE2*, which encodes an error–prone DNA polymerase, was shown to increase its expression in response to DNA damaging agents, suggesting that its role is involved in damage tolerance [33,34]. mRNA levels of *M*. *smegmatis dnaE2* and *recA* were increased 46-fold and 12-fold, respectively, by 0.2 mM H_2_O_2_, and 5-fold and 7.8-fold, respectively, by 7 mM H_2_O_2_ (Table 2). The response profiles with high inductions of DNA repair genes in *M*. *smegmatis* by both low (0.2 mM) and high (7 mM) levels of H_2_O_2_ were strikingly different to those in *M*. *tuberculosis* which showed high induction from mild levels of H_2_O_2_ and no change in induction with bactericidal H_2_O_2_ levels [7]_._ Future work should compare and investigate differences between *M*. *smegmatis* and *M*. *tuberculosis* in DNA-damage-mediated death caused by H_2_O_2_ in order to provide greater insights into the pathogenicity of *M*. *tuberculosis*. The STRING database was used to establish protein interaction networks of physical and functional interactions among the differentially expressed genes identified (Fig 3B). Interestingly, using the KEGG-User Data Mapping [35] (Fig 4B), seven genes involved in fatty acid metabolism (RM018 and RM020) were found and formed an interconnected cluster (Table 2 and Fig 4A). In addition, nine genes involved in glycolysis/gluconeogenesis (msm00010) were found in a partially interconnected cluster (Table 2). Transcription of these genes was induced, suggesting that these differentially expressed genes are involved in the central carbon metabolism (CCM) switch and providing supporting evidence for a previous suggestion that the CCM of *M*. *tuberculosis* plays important roles in growth and pathogenicity [36,37]. An extensive transcriptional switch in *M*. *tuberculosis* CCM genes during host infection has been reported, indicating that there is a quick change in the metabolic pathway in response to various stresses [38]. The gene *pdhB* involved in glycolysis/gluconeogenesis was repressed 9.7-fold by 0.2 mM H_2_O_2_, and *pdhA* was repressed 11-fold. PdhA and PdhB are constituents of the mycobacterial pyruvate dehydrogenase complex which connects glycolysis and the tricarboxylic acid (TCA) cycle [39]. In contrast to cells treated with 7 mM H_2_O_2_, changes induced in *pdhA* and *pdhB* in the 0.2 mM treatment were weak (Table 2). Similarly, the seven differentially expressed genes induced by 0.2 mM were involved in fatty acid metabolism and decreased by 4 to 6-fold (Table 2), whereas a decrease induction did not appear after the 7 mM H_2_O_2_ treatment. Together, these results suggest that the metabolic switch of glycolysis/gluconeogenesis and fatty acid metabolism was specific to induction by 0.2 mM H_2_O_2_.

**Table 2:** Fold changes of genes differentially expressed after treatment with 0.2 mM and 7 mM H_2_O_2_ (treated vs untreated).

Gene Name	Gene Product	log2 Ratio (0.2mM/mc^2^155)	P-value	FDR	log2 Ratio (7mM/mc^2^155)	P-value	FDR
**DNA repair**							
*msmeg_2839*	transcriptional accessory protein	-1.7523338	2.02E-243	5.7E-242	-1.6975845	9.0323E-80	5.4177E-79
*alkA*	methylated-DNA—protein-cysteine methyltransferase	-2.106759	8.54E-09	2.73E-08	-1.1090648	0.00020253	0.00030498
*msmeg_1383*	endonuclease IV	-2.8849149	0	0	-0.4418784	0	0
*msmeg_1756*	endonuclease VIII and DNA N-glycosylase with an AP lyase activity	3.01822392	2.08E-105	3.04E-104	2.96577039	9.82E-104	7.22E-103
*dnaE2*	error-prone DNA polymerase	5.52502439	0	0	2.44964676	1.32E-277	2.48E-276
*lig*	ATP-dependent DNA ligase	2.37386688	1.03E-184	2.21E-183	1.60677746	5.66E-69	3E-68
*ligA*	NAD-dependent DNA ligase LigA	2.32734432	0	0	2.47575753	0	0
*recA*	recombinase A	3.59091497	0	0	2.96402929	0	0
*uvrB*	excinuclease ABC subunit B	3.25788081	0	0	4.50884994	0	0
*lexA*	LexA repressor	3.10981267	0	0	1.01678782	4.81E-103	3.51E-102
*radA*	DNA repair protein RadA	2.82617738	0	0	1.71244303	0	0
*dinP*	DNA polymerase IV	4.26301055	1.85E-174	3.84E-173	4.18732816	2.79E-169	3.17E-168
*msmeg_1622*	putative DNA repair polymerase	4.79317887	8.44E-277	2.71E-275	-0.1339395	0.658812	0.69126208
*recD*	exodeoxyribonuclease V, alpha subunit	3.50057135	3.24E-216	8.07E-215	2.03125644	2.04E-46	8.42E-46
*msmeg_1756*	endonuclease VIII and DNA N-glycosylase with an AP lyase activity	3.01822392	2.08E-105	3.04E-104	2.96577039	9.82E-104	7.22E-103
*helicase*	ATP-dependent DNA helicase	4.16679804	0	0	4.74283172	0	0
*tag*	DNA-3-methyladenine glycosylase I	2.81384991	2.09E-184	4.47E-183	2.86173411	2.34E-201	3.15E-200
**Glycolysis/gluconeogenesis**							
*msmeg_1543*	eptc-inducible aldehyde dehydrogenase	-2.6859775	8.91E-139	4.21E-148	-1.2892582	5.64E-56	2.60E-55
*msmeg_1762*	piperideine-6-carboxylic acid dehydrogenase	2.93235592	2.30E-149	4.21E-148	-1.6876011	2.28E-12	4.83E-12
*pfkB*	6-phosphofructokinase isozyme 2	-5.7653785	3.88E-196	8.90E-195	-2.8501465	6.61E-126	5.73E-125
*pdhB*	pyruvate dehydrogenase E1 component subunit beta	-3.2722769	3.01E-30	1.92E-29	0.71595693	5.59E-07	9.61E-07
*pdhA*	pyruvate dehydrogenase E1 component, alpha subunit	-3.4705711	1.84E-89	2.39E-88	-0.3895867	0.00010596	0.00016157
*adhB*	alcohol dehydrogenase B	-4.0478653	2.97E-105	4.33E-104	-2.1612025	4.92E-60	2.38E-59
*msmeg_6616*	S-(hydroxymethyl)glutathione dehydrogenase	-2.956082	0.00E+00	0.00E+00	-1.1422205	1.36E-159	1.45E-158
*msmeg_6687*	aldehyde dehydrogenase, thermostable	-3.706221	1.64E-29	1.03E-28	-1.5049935	9.36E-12	1.94E-11
*msmeg_6834*	alcohol dehydrogenase	-2.7848309	2.21E-05	5.51E-05	0.08733237	0.840712	0.85579383
*fadA*	acetyl-CoA acetyltransferase	-2.7273404	0.00E+00	0.00E+00	0.33348045	5.36E-35	1.86E-34
*fadB*	putative 3-hydroxyacyl-CoA dehydrogenase	-2.6949142	0.00E+00	0.00E+00	1.39743214	0	0
*msmeg_5199*	putative acyl-CoA dehydrogenase	-2.6174798	7.78E-91	1.02E-89	-0.4351779	5.88E-07	1.01E-06
*msmeg_4832*	acyl-CoA dehydrogenase	-2.2726247	1.84E-34	1.30E-33	-1.4448556	2.45E-20	6.42E-20
*msmeg_4833*	putative acyl-CoA dehydrogenase	-2.176661	8.50E-49	7.39E-48	-2.3488919	3.50E-57	1.65E-56
*echA4*	enoyl-CoA hydratase	-2.5812975	1.27E-32	8.59E-32	-0.1876747	0.1805012	0.20801109
*fadE13*	putative acyl-CoA dehydrogenase	-2.4369076	3.84E-11	1.38E-10	-0.1310912	0.579156	0.6139111
**Sigma factors**							
*sigG*	RNA polymerase factor sigma-70	2.93398738	0	0	3.88123043	0	0
*msmeg_0573*	putative ECG sigma factor RpoE1	1.38509413	0.0569946	0.08901435	7.09387521	1.35E-209	1.91E-208
*msmeg_0574*	putative ECG sigma factor RpoE1	0.18840222	0.422278	0.5067336	4.08443636	3.1E-276	5.78E-275
*msmeg_1348*	RNA polymerase ECF-subfamily protein sigma factor	-0.0465176	0.804554	0.84797939	-2.3586787	1E-17	2.48E-17
*sigL*	RNA polymerase sigma factor SigL	0.52825986	0.00083713	0.00176811	-2.2355973	4.27E-20	1.12E-19
*msmeg_1970*	sigma factor	-1.6126499	4.2715E-20	1.1152E-19	-4.1999183	4.27E-20	1.12E-19
*mysB*	RNA polymerase sigma factor SigB	-0.4921846	4.74E-104	6.85E-103	2.50111799	0	0
*msmeg_3008*	putative sigma 54 type regulator	-1.8964762	1.61E-11	5.87E-11	-2.0197975	3.44E-13	7.46E-13
*msmeg_5214*	RNA polymerase sigma-70 factor	4.65312922	2.09E-184	4.47E-183	2.81376729	1.63E-13	3.59E-13
*msmeg_5444*	RNA polymerase sigma-70 factor protein	0.03717083	0.894318	0.91679688	-2.0919913	9.72E-09	1.8E-08
**Transcriptional Regulators**							
*devR*	two-component system response regulator	1.01514452	0.00136283	0.00280756	6.64431111	0	0
*furA (msmeg_6383)*	transcription regulator FurA	2.0393827	1.57E-38	1.19E-37	4.16725591	0	0
*phoP*	DNA-binding response regulator PhoP	2.90191537	4.67E-220	1.19E-218	-0.5325639	0.00089158	0.00128064
*msmeg_4517*	TetR-type transcriptional regulator of sulfur metabolism	3.21516913	0.00000169	0.00000457	4.13040109	7.31E-14	1.62E-13
*msmeg_4925*	transcriptional regulator	0.69321643	0.00330216	0.00644626	2.93688979	1.02E-78	6.03E-78
*msmeg_1919*	Transcription factor WhiB	2.52297686	0	0	1.07820136	2.53E-126	2.21E-125
*msmeg_4025*	transcriptional regulator, LysR family protein	1.30263197	0.00055968	0.00120459	5.38130323	1.6E-210	2.28E-209
*msmeg_6253*	fur family protein transcriptional regulator	1.24070422	0.0469844	0.07479434	3.90800867	1.22E-25	3.59E-25
**Detoxification enzymes**							
*trxB*	thioredoxin-disulfide reductase	-0.0780198	0.1478068	0.20755437	2.71109242	0	0
*trx*	thioredoxin	0.12205973	0.0602136	0.09347541	3.84889994	0	0
*msmeg_6884*	NADP oxidoreductase	0.87052096	0.1166366	0.1680318	4.68101209	4.52E-68	2.38E-67
*KatG*	catalase/peroxidase HPI	3.82587233	0	0	4.95397834	0	0
*msmeg_4890*	alkylhydroperoxidase	3.42735043	6.73E-19	3.21E-18	4.22476909	0.0465926	0.05777446
*msmeg_3448*	two-component system sensor kinase	0.55015338	0.1588256	0.22100457	5.07235098	4.09E-202	5.55E-201
*ahpD*	alkylhydroperoxidase AhpD core	-2.3697934	0.0210456	0.03618406	3.85029317	4.71E-31	1.53E-30
*msmeg_3708*	catalase	2.47422002	5.45E-56	5.11E-55	-3.0001305	5.73E-15	1.31E-14

Compared to the down-regulation of 331 genes under the 0.2 mM H_2_O_2_ treatment, 1671 genes were down-regulated under the 7 mM H_2_O_2_ treatment and 343 genes were up-regulated. In contrast to the small proportion of genes in the genome that responded to the 0.2 mM H_2_O_2_ treatment (663 differentially-expressed genes, ~10% of the genes in the *M*. *smegmatis* genome), 2002 genes were induced in response to 7 mM H_2_O_2_, representing 29.3% of the genes in the *M*. *smegmatis* genome. In contrast to the interaction networks obtained among genes which showed differential expression at the mRNA level in response to the 0.2mM H_2_O_2_ treatment, we did not find enrichment in specific metabolic pathways among genes that were differentially expressed in response to the 7 mM H_2_O_2_ treatment. This might be due to the fact that the 7 mM H_2_O_2_ treatment had more global effects on transcription, which were not limited to specific metabolic pathways. We also conducted an analysis of GO biological processes and identified enrichment in processes including gene expression (p = 2.47 x 10^−1^), macromolecule biosynthetic processes (p = 2.47 x 10^−1^) and regulation of gene expression (p = 2.84 x 10^−1^) (S1 Fig). Ribosome biogenesis was also enriched, though the P-value was 5.48 x 10^−1^, slightly higher than the cutoff value (P < 0.5). Oxidative stress results in the rapid inhibition of protein synthesis as well as in the reprogramming of gene expression, resulting in growth reduction as an adaption to oxidative stress [40].

### Responses of specific genes to exposure to low and high levels of H_2_O_2_

*Mycobacterium* utilizes diverse antioxidant systems to combat H_2_O_2_ stress, including sigma factors, transcriptional regulators, STKs and detoxifying enzymes. To further clarify the responses to different levels of H_2_O_2_ stress, we compared the biological categories involved in oxidative stress. In *M*. *tuberculosis*, sigma factors (such as SigE, SigH, SigL and SigF) are important for initiating the H_2_O_2_ detoxification pathway [7], and so we examined the response of sigma factors to different levels of H_2_O_2_ in *M*. *smegmatis*. We did not find any differences in the expression of *sigF* in the 0, 0.2 mM, and 7 mM H_2_O_2_ treatments. SigF was first described as a stationary-phase stress response sigma factor in *M*. *tuberculosis* [41], and we have previously shown that SigF is involved in the oxidative stress response in mycobacteria [41,42]. Lack of SigF induction here may have been due to the fact that SigF is a stationary-phase stress response sigma factor and does not function at the early logarithmic phase, which was used in this study. It will be interesting to compare the global transcriptional response to H_2_O_2_ at different growth phases. While Voskuil et al. conducted studies showing that *M*. *tuberculosis* SigH is highly induced upon exposure to high levels of H_2_O_2_ [7], here, *M*. *smegmatis* SigH was moderately induced at both low and high stresses (data not shown). The gene *msmeg_0573*, which encodes the ECF sigma factor RpoE1, was the most highly induced sigma factor, exhibiting a 136-fold increase in expression following the 7 mM H_2_O_2_ treatment. No change in its expression was observed following the 0.2 mM H_2_O_2_ treatment. Its paralog, *msmeg_0574*, which encodes the ECF sigma factor RpoE1, was also specifically induced (~17-fold) by 7 mM H_2_O_2_. These results showed that both MSMEG_0573 and MSMEG_0574 are specifically induced by 7 mM H_2_O_2_. In contrast, *msmeg_5214*, which encodes the RNA polymerase sigma-70 factor, was specifically up-regulated in 0.2 mM H_2_O_2_ (Table 2). Together, these data indicate that distinct sigma factor genes respond to different levels of H_2_O_2_. In addition to sigma factors, transcriptional regulators also have important roles in the regulation of the oxidative stress response. As *M*. *tuberculosis* does not have a functional OxyR, the transcription regulators FurA, IdeR, CarD and WhiB play important roles in the oxidative stress response. FurA was induced 4-fold at 0.2 mM H_2_O_2_ and 18-fold at 7 mM H_2_O_2_. Furthermore, no significant changes were found in *IdeR* and *CarD* expression levels at either 0.2 mM or 7 mM H_2_O_2._ The *M*. *smegmatis* genome contains six *whiB* genes, and only MSMEG_1919 was induced by 0.2 mM H_2_O_2_ (Table 2). DevR has previously been shown to be highly induced in *M*. *tuberculosis* only when exposed to high concentrations of H_2_O_2_ [7]. Consistent with this report, our results showed that in *M*. *smegmatis*, *devR* expression was mildly increased by 0.2 mM and strongly increased (100-fold) after treatment with 7 mM H_2_O_2_ treatment (Table 2). We next compared the transcription responses of genes coding for serine/threonine-protein kinases (SPTKs) to H_2_O_2_ exposure. Of the STPKs, we found that following 7 mM H_2_O_2_ treatment, only *pknK* was up-regulated. *M*. *tuberculosis* PknK has been shown to regulate the slow growth of mycobacteria in response to various stresses and during persistence in infected mice [43]. Just as PknK is involved in the oxidative stress response pathway in *M*. *tuberculosis*, PknK plays an important role in *M*. *smegmatis* in regulating the response to high levels of H_2_O_2_. The functions of PknK and its involvement in the response to H_2_O_2_ require further exploration.

The *M*. *smegmatis* genome encodes several enzymes involved in the detoxification of H_2_O_2_ [4,8]. Our analysis showed that mRNA levels of *katG* were up-regulated 12-fold and 30-fold following exposure to 0.2 and 7 mM H_2_O_2_ respectively (Table 2). Notably, *trxB* (encoding thioredoxin-disulfide reductase) and *trx* (encoding thioredoxin) expression was induced strongly by 7 mM H_2_O_2_ treatment (Table 2), but not by 0.2 mM H_2_O_2_. Similarily, *ahpD* (encoding alkylhydroperoxidase) and *msmeg_6884* (encoding NADP oxidoreductase) responded to 7 mM H_2_O_2_ but not to 0.2 mM H_2_O_2_. *msmeg_3708* (encoding catalase) exhibited a 5-fold increase in mRNA expression only after exposure to 0.2 mM H_2_O_2_, indicating that it is specifically induced in response to low levels of H_2_O_2_ exposure. It will be interesting to investigate the distinct biological roles of enzymes that scavenge different levels of H_2_O_2_ in mycobacteria. Such studies will lead to greater understanding of the basic roles of these enzymes.

## Conclusion

In this study, we have shown that, in *M*. *smegmatis*, different genes are induced in response to low and high levels of H_2_O_2_. A notable difference in the response to low-level (0.2 mM) H_2_O_2_ and high-level (7 mM) H_2_O_2_ was observed. When exposed to 0.2 mM H_2_O_2_, expression of approximately 10% of the genes in the *M*. *smegmatis* genome was significantly changed. In contrast, 29.3% of *M*. *smegmatis* genes were significantly changed in response to 7 mM H_2_O_2._ Transcriptional analysis suggested that a metabolic switch in glycolysis/gluconeogenesis and fatty acid metabolism was potentially involved in the response to the 0.2 mM H_2_O_2_ treatment but not to the 7 mM H_2_O_2_ treatment. We also observed that transcriptional levels of genes encoding ribosomes decreased when bacterial cells were treated with 7 mM H_2_O_2_. This result suggests that 7 mM H_2_O_2_ treatment affected the protein synthesis apparatus and thus reduced protein synthesis, resulting in reduced bacterial growth. The expression level of gene *msmeg_5214* (encoding the RNA polymerase sigma-70 factor) was induced in response to 0.2 mM H_2_O_2_, and the *rpoE1*s (*msmeg_0573* and *msmeg_0574*) were induced specifically in response to 7 mM H_2_O_2_. In addition, different regulators were observed to respond to different levels of H_2_O_2_. MSMEG_1919 was induced following exposure to 0.2 mM H_2_O_2_, while DevR was highly induced by the 7 mM H_2_O_2_ treatment. Our results show that *pknK*, a gene encoding a STPK, is involved in the 7 mM H_2_O_2_ treatment response and that different genes encoding detoxifying enzymes, including the genes encoding KatG, AhpD, TrxB and Trx, were expressed in response to different levels of H_2_O_2_. In summary, this study of global transcriptional changes that occur after exposure to different levels of H_2_O_2_ documents changes in transcriptional regulation in response to exposure to low and high level H_2_O_2_ treatments, including the use of different sigma factors, regulators, serine/threonine kinases and differences in the transcriptional levels of detoxifying enzymes used to combat H_2_O_2_ stress. Further study of these genes will aid our understanding of the mechanisms underlying the precise regulation and scavenging of H_2_O_2_.

## Supporting Information

S1 Fig Overview of differential gene expression in response to 7 mM H_2_O_2_ in *M*. *smegmatis*. (JPG)

S1 Table Oligonucleotide primers used in this study. (DOC)

S2 Table RNA-sequencing expression data for the control of *M*. *smegmatis* mc^2^155 without H_2_O_2_ treatment. (XLSX)

S3 Table RNA-sequencing expression data for the *M*. *smegmatis* mc^2^155 strain treated with 0.2 mM H_2_O_2._
(XLSX)

S4 Table RNA-sequencing expression data for the *M*. *smegmatis* mc^2^155 strain treated with 7 mM H_2_O_2._
(XLSX)
