# Supplementary figures and images for: Distinct Responses of Mycobacterium smegmatis to Exposure to Low and High Levels of Hydrogen Peroxide
Source: PLoS One. 2015 Jul 30;10(7):e0134595. doi: 10.1371/journal.pone.0134595 (PMC4520597; doi:10.1371/journal.pone.0134595)

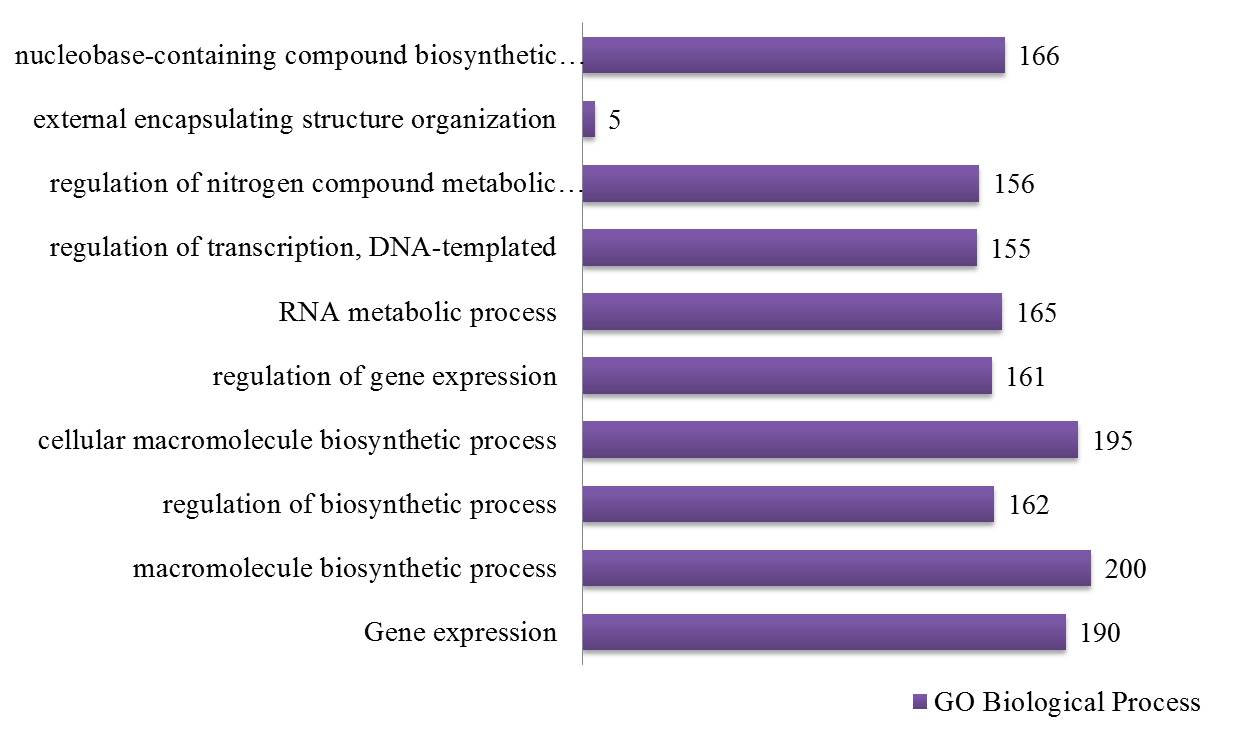

Supplement: S1 Fig — (JPG) [file pone.0134595.s001.jpg]
