# Supplementary material for: Distinct Responses of Mycobacterium smegmatis to Exposure to Low and High Levels of Hydrogen Peroxide
Source: PLoS One. 2015 Jul 30;10(7):e0134595. doi: 10.1371/journal.pone.0134595 (PMC4520597; doi:10.1371/journal.pone.0134595)
Supplement: S1 Table — (DOC) [file pone.0134595.s002.doc]

**Table S1. Oligonucleotide primers used in this study**

| Name | 5’-3’ | purpose |
| --- | --- | --- |
| Msm0574qF | GTGCAGGAGACGATGGTCAA | qRT-PCR |
| Msm0574qR | CCACGTGTTCGTGAGGATCT |
| MsmRpoDqF | GTGTGGGACGAGGAAGAGTC | qRT-PCR |
| MsmRpoDqR | ACCTCTTCTTCGGCGTTGAG |
| Msm1583qF | TCCTCGACGTTGGAGACCTA | qRT-PCR |
| Msm1583qR | GTCGAAGTCGGTGACGAAGT |
| Msm3242qF | CCAAGGTTTCCATGCCACAC | qRT-PCR |
| Msm3242qR | GTTCCAATGGGCCTGTTTGC |
| Msm3447qF | CCAACACGCCAAAGAGCTTC | qRT-PCR |
| Msm3447qR | GATTGCGTCGAGCAACACAC |  |
| Msm5214qF | GCTGAGGTCATGGCTGTTCA | qRT-PCR |
| Msm5214qR | CGGAGTGTCAGGGGATGAAA |  |
| Msm1804qF | CGGCTCAAGGAACTCCACTT | qRT-PCR |
| Msm1804qR | TCTTCGCGATCCATGTCCAG |  |
| Msm0529qF | CGCAGGCAGAACCTCGGCAA | qRT-PCR |
| Msm0529qR | GGCCATCACCACGCGTTCGT |  |
| Msm1486qF | CGTGGCACGCAACATGATTA | qRT-PCR |
| Msm1486qR | GTATCCACCTGGTCGGAGGT |  |
| Msm1919qF | GTGACCACCGAATGCCTGA | qRT-PCR |
| Msm1919qR | CGTTGCGACGCTTGAGTG |  |
| Msm4298qF | GTTCGGCAGCTATGAGGACG | qRT-PCR |
| Msm4298qR | ATCTGATCAGCGACACGCTC |  |
| Msm4514qF | GGAGTCCGAGATCATCGCC | qRT-PCR |
| Msm4514qR | TTGAACGCCTCGTAGTCGG |  |
| Msm4891qF | GGACAACGAGTTCGTCCACT | qRT-PCR |
| Msm4891qR | AGATCCGAGACCATCGGGAA |  |
| Msm5575qF | GCATCGCGAGTTGTCGTG | qRT-PCR |
| Msm5575qR | GATGGTTTCCATGCCGGTCT |  |
